# Distinct prognostic impact of diabetes subtypes, metformin use, and glycemic control in pancreatic cancer: a systematic review and meta-analysis of cohort studies

**DOI:** 10.3389/fonc.2026.1903503

**Published:** 2026-08-06

**Authors:** Guangxin Li, Zha Peng, Yaqiong Wang, Zhuangrong Zhu, Shengquan Huang, Jiangsan Yu, Hai Huang

**Affiliations:** 1 The Wuming Hospital of Guangxi Medical University, Nanning, Guangxi, China; 2 Department of Surgery, Affiliated Dongguan Hospital, Guangzhou University of Chinese Medicine, Dongguan, Guangdong, China

**Keywords:** diabetes subtypes, hyperglycemia, meta-analysis, metformin, pancreatic cancer

## Abstract

**Background:**

The prognostic significance of glycemic status in pancreatic ductal adenocarcinoma (PDAC) remains controversial, largely due to heterogeneity in the exposure definitions (new-onset *vs*. long-standing diabetes, metformin use, and single fasting glucose measurement). This meta-analysis aimed to clarify the association of four distinct glycemic exposures with overall survival (OS) in patients with PDAC.

**Methods:**

We systematically searched Web of Science, PubMed, and EMBASE from 1 January 2010 to 1 June 2026. Cohort studies reporting hazard ratios (HRs) for OS were included and categorized into four subgroups: 1) new-onset diabetes (NOD) *vs*. non-diabetes; 2) long-standing diabetes (LSD) *vs*. non-diabetes; 3) metformin use *vs*. non-use; and 4) hyperglycemia (elevated preoperative fasting glucose or poor glycemic control) *vs*. normoglycemia. Random-effects meta-analyses were performed. Heterogeneity was assessed using *I*^2^, and publication bias was evaluated via funnel plots and the trim-and-fill method.

**Results:**

A total of 21 studies (six for NOD, four for LSD, six for metformin, and eight for hyperglycemia) were included. NOD was significantly associated with worse OS (pooled HR = 1.33, 95%CI = 1.13–1.57, *I*^2^ = 72.5%). LSD showed no significant association (HR = 1.21, 95%CI = 0.98–1.49, *I*^2^ = 70.1%). Metformin use was associated with a 33% reduction in mortality (HR = 0.67, 95%CI = 0.53–0.84, *I*^2^ = 70.5%). Preoperative hyperglycemia or poor glycemic control consistently predicted worse OS (HR = 1.45, 95%CI = 1.29–1.62, *I*^2^ = 25.7%). No substantial publication bias was detected (trim-and-fill added zero studies for all subgroups).

**Conclusions:**

NOD and preoperative hyperglycemia are independent negative prognostic factors in PDAC, whereas LSD did not show a statistically significant association. An indirect comparison between NOD and LSD yielded a null result, suggesting that diabetes duration may not be the key determinant of prognosis. The potential mediating role of hyperglycemia requires further investigation. Metformin use was associated with a marked survival benefit, particularly in patients with resectable tumors. These findings highlight the need to differentiate diabetes subtypes and optimize glycemic control, while clinical decisions regarding antidiabetic agents should be made with caution.

**Systematic Review Registration:**

https://www.crd.york.ac.uk/prospero/display_record.php?RecordID=261415381, identifier CRD420261415381.

## Introduction

Pancreatic ductal adenocarcinoma (PDAC) is one of the most lethal malignancies, with a 5-year survival rate below 10% (1). Despite advances in surgical techniques and chemotherapy, the prognosis remains dismal, partly due to late diagnosis and aggressive tumor biology (2, 3). Identifying modifiable prognostic factors is critical to improving outcomes.

Diabetes mellitus (DM) is highly prevalent in PDAC, affecting up to 40%–60% of patients (4). However, the relationship between DM and PDAC survival is complex and bidirectional. New-onset diabetes (NOD) often arises as a paraneoplastic phenomenon within 2 years (or up to 3 years in some studies) before PDAC diagnosis, whereas long-standing diabetes (LSD) is a preexisting risk factor (5–7). In addition, metformin, a first-line antidiabetic drug, has been suggested to exert direct antitumor effects, while hyperglycemia itself may promote tumor progression through metabolic reprogramming and activation of oncogenic pathways (8, 9).

Previous meta-analyses have yielded conflicting results, largely because they treated “diabetes” as a single exposure and did not differentiate NOD from LSD, nor did they separately examine metformin or single fasting glucose levels (8–10). Even recent meta-analyses pooling up to 80 cohort studies still treated diabetes uniformly without subtype differentiation (10). For instance, Wan et al. reported a significant survival benefit of metformin adjuvant treatment in patients with pancreatic cancer [hazard ratio (HR) = 0.88, 95%CI = 0.80–0.97]; however, their analysis did not distinguish between the different diabetic subtypes or the glycemic control status (11). Therefore, we conducted a systematic review and meta-analysis to evaluate the prognostic impact of four distinct glycemic exposures: NOD, LSD, metformin use, and preoperative hyperglycemia (including poor glycemic control).

## Methods

### Search strategy and study selection

This study followed the Preferred Reporting Items for Systematic Reviews and Meta-Analyses (PRISMA) 2020 guidelines. We systematically searched PubMed, EMBASE, and Web of Science from 1 January 2010 to 1 June 2026. This start date was chosen because 2010 marked the widespread adoption of HbA1c ≥6.5% as a diagnostic criterion for diabetes by the American Diabetes Association, which standardized the definition of diabetes across contemporary cohort studies and enabled consistent categorization of NOD *versus* LSD in relation to PDAC.

The search strategy combined terms for pancreatic cancer, glycemic status, and survival. The detailed search strings for each database are provided in **Supplementary Table 1**.

This systematic review was prospectively registered in PROSPERO (CRD420261415381, available at: https://www.crd.york.ac.uk/prospero/display_record.php?RecordID=261415381..

Records from PubMed, EMBASE, and Web of Science were screened after removal of duplicates. The screening process was conducted in three stages, as follows: 1) title/abstract screening to exclude irrelevant records; 2) eligibility screening based on exposure definitions and outcome reporting to select full-text articles; and 3) full-text review for final inclusion. Cohort studies that met all inclusion criteria were included in the quantitative synthesis.

### Inclusion criteria

Cohort studies (prospective or retrospective) with pathologically confirmed PDAC were included. The exposures had to be clearly defined as one of the following: NOD, diagnosed ≤24 months before PDAC; LSD, primarily defined as diagnosed >24 months before PDAC (noting that Jeon et al. used a >3-year threshold and Tang et al. did not specify duration, as detailed in the footnotes in **Table 1**) (12, 13); metformin use (*vs*. non-use); or hyperglycemia (preoperative fasting glucose >6.1 mmol/L, HbA1c >6.5%, or study-defined poor glycemic control). Eligible studies were required to report an HR with a 95% confidence interval (CI) for overall survival (OS) or provide sufficient data for their calculation. Only English-language publications were considered.

**Table 1:** Characteristics of the included studies.

Study	Year	Country	Design	Sample size	Age (years)^a^	Sex (male %)^b^	Tumor stage	Exposure definition	Comparator	Main adjustment factors	NOS score
New-onset diabetes (NOD) subgroup											
Lee S (22)	2018	Republic of Korea	Retrospective cohort	266	61–65 (range)	57	Resectable (I–II)	NOD (≤24 months)	Non-diabetes	T stage, N stage, CA19-9	7
Liu PJ (21)	2025	China	Multicenter retrospective	1,211	NR	NR	Resectable (R0)	NOD (≤24 months)	Non-diabetes	Age, BMI, tumor size, differentiation, complications	8
Li Y (15)	2025	China	Retrospective cohort	1,620	60 (median)	60.6	Mixed (I–IV)	NOD (≤2 years)	Non-diabetes	Age, TNM stage, treatment	7
Mohindroo C^c^ (14)	2025	USA	Retrospective cohort	138	70 (median)	47	Locally advanced/metastatic	NOD (≤3 years)	Non-diabetes	Univariate (estimated)	6
Jeon CY^d^ (12)	2018	Multi-country	Pooled analysis	2,792	63.3 (mean)	55	Resectable/locally advanced/metastatic	NOD (≤3 years)	Non-diabetes	Age, sex, stage, smoking, BMI, study site	8
Kanbour S^e^ (20)	2023	USA	Retrospective cohort	1,556	66.4 (mean)	51	Resectable	NOD (≤2 years)	Non-diabetes	Age, sex, stage, tumor location, margin, etc.	8
Long-standing diabetes (LSD) subgroup											
Liu PJ (21)	2025	China	As above	1,211	NR	NR	As above	LSD (>24 months)	Non-diabetes	Multivariable	8
Jeon CY^d^ (12)	2018	Multi-country	As above	2,792	63.3 (mean)	55	As above	LSD(>3 years)	Non-diabetes	Age, sex, stage, smoking, BMI, study site	8
Kanbour S (20)	2023	USA	As above	1,556	66.4 (mean)	51	As above	LSD (≥2 years)	Non-diabetes	Age, sex, stage, tumor location, margin	8
Tang Z¶ (13)	2022	China	Retrospective cohort	238	NR	55	Early stage (I–II)	LSD (T2DM, duration not specified)	Non-diabetes	Sex, tumor differentiation	7
Metformin subgroup											
Lee SH (23)	2016	Republic of Korea	Retrospective cohort	237	66 (median)	66.2	Mixed	Metformin use (≥1 month)	No metformin	CA19-9, stage, performance status	8
Dulskas A (24)	2020	Lithuania	Retrospective cohort (registry)	454	NR	NR	Mixed	Metformin use	No metformin	Age, sex, stage, histology	7
Terasaki F (26)	2020	Japan	Retrospective cohort	121	NR	65	Resectable	Metformin use (preoperative)	Other hypoglycemics	pN, vascular invasion, CA19-9	7
Takahashi R (25)	2025	Japan	Retrospective cohort	65	NR	NR	Resectable	Metformin use	No metformin	Propensity score matched	7
Wynn A (16)	2019	USA	Retrospective cohort	46	NR	95 (VA cohort)	Mixed	Metformin use (≥6 months)	No metformin	Univariate (estimated)	6
Chaiteerakij R (27)	2016	USA	Retrospective cohort	980	67.4 (mean)	58.6	Mixed	Metformin use (time-varying)	No metformin	Age, sex, BMI, stage, diagnosis year	8
Hyperglycemia/poor glycemic control subgroup											
Toriola AT (29)	2014	USA	Prospective cohort	504	64 (median)	53.6	Local/locally advanced/metastatic	History of diabetes	No diabetes	Age, sex, BMI, race, smoking, stage	8
Zhang M (28)	2021	China	Retrospective cohort	253	60 (median)	58.5	Mixed	FBG >6.11 mmol/L	FBG ≤6.11	CA19-9, back pain	7
Iarrobino NA (30)	2019	USA	Retrospective cohort	303	70 (median)	50.2	Locally advanced	Max glucose ≥200 mg/dL	<200 mg/dL	Age, surgery, chemotherapy	7
Lee HJ (34)	2026	Republic of Korea	Retrospective cohort	255	64–65 (median)	~60	Mixed	HbA1c ≥8%	HbA1c <8%	Age, sex, stage, treatment	7
Wang S (31)	2025	China	Retrospective cohort	225	61–64 (median)	66.7	Mixed	FBG >6.11 mmol/L	FBG ≤6.11	Age, CA19-9	7
Kubo H (32)	2025	Japan	Retrospective cohort	569	69 (median)	59	Resectable	Preoperative HbA1c ≥9.0%	<9.0%	pN, metformin, CA19-9, age	7
Alpertunga I (17)	2021	USA	Retrospective cohort (biobank)	73	72 (median)	52	Locally advanced/metastatic	RBG-3 >120 mg/dL	≤120 mg/dL	Multivariable (inverted)	6
Zhu X (33)	2023	China	Retrospective cohort (prospective database)	697	NR	63.7	Locally advanced/metastatic	FBG ≥7.0 mmol/L (no known DM)	FBG <7.0	Age, sex, location, size, metastasis, CA19-9, treatment	7

*NR*, not reported; *FBG*, fasting blood glucose; *T2DM*, type 2 diabetes mellitus; *NOS*, Newcastle–Ottawa Scale.

^a^ Age presented as median (range or IQR) or mean ± SD where reported.

^b^ Sex presented as percentage of male participants. Where available, exact numbers can be found in the original publications.

^c^ Mohindroo et al. (14) defined NOD as ≤3 years.

^d^ Jeon et al. (12) defined NOD as ≤3 years and LSD as >3 years, deviating from the 24-month threshold.

^e^ Kanbour et al. (20) defined LSD as ≥2 years (approximately equivalent to >24 months).

^f^ Tang et al. (13) did not specify the diabetes duration; included with caution.

### Exclusion criteria

Case reports, cross-sectional studies, randomized controlled trials without cohort data, duplicate publications, and studies without extractable survival data were excluded.

### Data extraction and quality assessment

Two reviewers independently extracted the following information: first author, year, country, study design, sample size, exposure definition, comparator, HR (preferably adjusted), 95%CI, adjustment factors, and follow-up duration. Quality was assessed using the Newcastle–Ottawa Scale (NOS), with a maximum score of 9 stars (**Supplementary Table 2**). The NOS scores were interpreted as follows: 0–3 = low quality, 4–6 = moderate quality, and 7–9 = high quality.

### Statistical analysis

For each exposure subgroup, we performed a random-effects meta-analysis using the DerSimonian–Laird method, implemented in the meta package of R (version 4.4.1). Heterogeneity was quantified using the *I*^2^ statistic, with values of 0%–25% indicating low heterogeneity, 26%–50% moderate, 51%–75% high, and 76%–100% very high heterogeneity. Publication bias was assessed primarily by visual inspection of funnel plots. Egger’s test was also performed for exploratory purposes; however, its results were interpreted with caution given the small number of studies per subgroup (*k* < 10), as recommended in the Cochrane Handbook. The trim-and-fill method was applied to adjust for potential missing studies. Sensitivity analyses (leave-one-out) were conducted for each subgroup. In addition, to explore the impact of variable temporal definitions of NOD and LSD, we conducted a *post-hoc* sensitivity analysis restricted to studies that strictly adhered to the 24-month cutoff for both NOD (≤24 months) and LSD (>24 months), excluding studies that used the 3-year definition (i.e., Mohindroo et al. and Jeon et al.) (12, 14). For studies that did not directly report HRs (14–17), we estimated HRs using established methods (18, 19) as detailed in **Supplementary Table 2**. Given the small number of studies in each subgroup (*k* = 4–8), we acknowledge that the DerSimonian–Laird random-effects model may underestimate uncertainty under these conditions. We therefore interpreted our pooled estimates with caution, particularly for subgroups with only four studies.

## Results

### Study selection and characteristics

**Figure 1:**
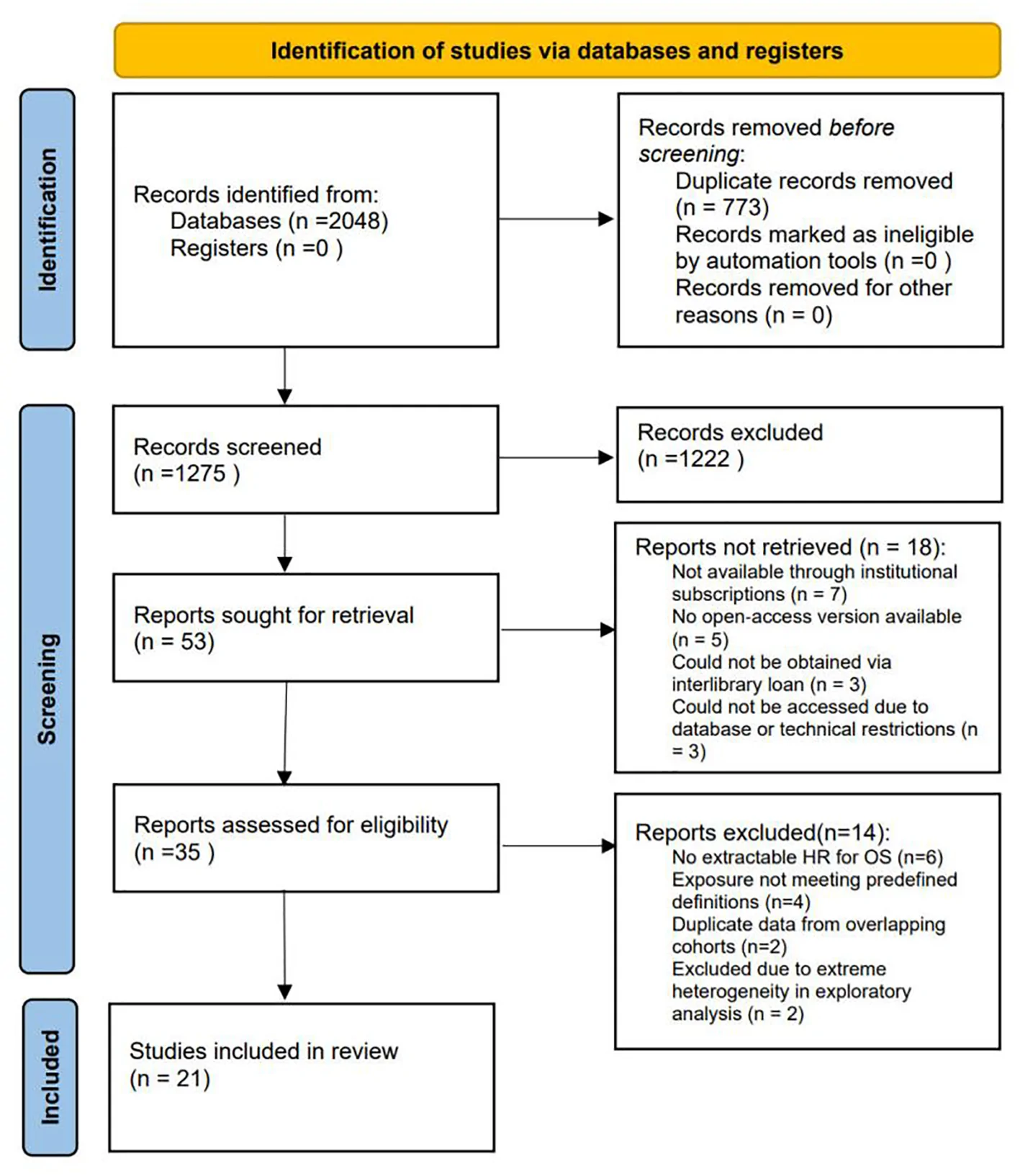
Preferred Reporting Items for Systematic Reviews and Meta-Analyses (PRISMA) 2020 flow diagram summarizing the study selection process.

The PRISMA flow diagram (**Figure 1**) summarizes the study selection process. The initial search yielded 2,048 records from the databases. After removal of duplicates, 1,275 unique records were screened, of which 1,222 were excluded during the title/abstract screening. The remaining 53 reports were sought for retrieval, of which 18 could not be accessed despite attempts through institutional subscriptions, open-access searches, interlibrary loan requests, and database access verification: seven were not available through our institutional subscriptions, five had no open-access version available, three could not be obtained despite interlibrary loan requests, and three could not be accessed due to database or technical restrictions. The remaining 35 full-text articles were assessed for eligibility, of which 14 were excluded for the following reasons: no extractable HR for OS (*n* = 6), exposure not meeting predefined definitions (*n* = 4), duplicate data from overlapping cohorts (*n* = 2), and extreme heterogeneity/outlier status in the exploratory analysis (*n* = 2). Consequently, 21 cohort studies published between 2010 and 2026 met all the inclusion criteria and were included in the quantitative synthesis, comprising a total of 10,488 patients with PDAC. The number of studies per subgroup was as follows: *n* = 6 for NOD, *n* = 4 for LSD, *n* = 6 for metformin use, and *n* = 8 for hyperglycemia. The total across subgroups (6 + 4 + 6 + 8 = 24) exceeded the 21 unique studies because three studies (Liu et al., Jeon et al., and Kanbour et al.) (12, 20, 21) contributed to both the NOD and LSD subgroups. The sample sizes across the studies ranged from 46 to 2,792 patients. Geographically, the included studies were conducted in East Asia (*n* = 12, predominantly China, Republic of Korea, and Japan), North America (*n* = 8, United States and multi-country pooled analyses), and Europe (*n* = 1, Lithuania). The NOS scores ranged from 6 to 8 (median = 7), indicating moderate to high methodological quality. Detailed characteristics of each included study are presented in **Table 1**.

### Meta-analysis results

#### New-onset diabetes *vs*. non-diabetes

**Figure 2:**
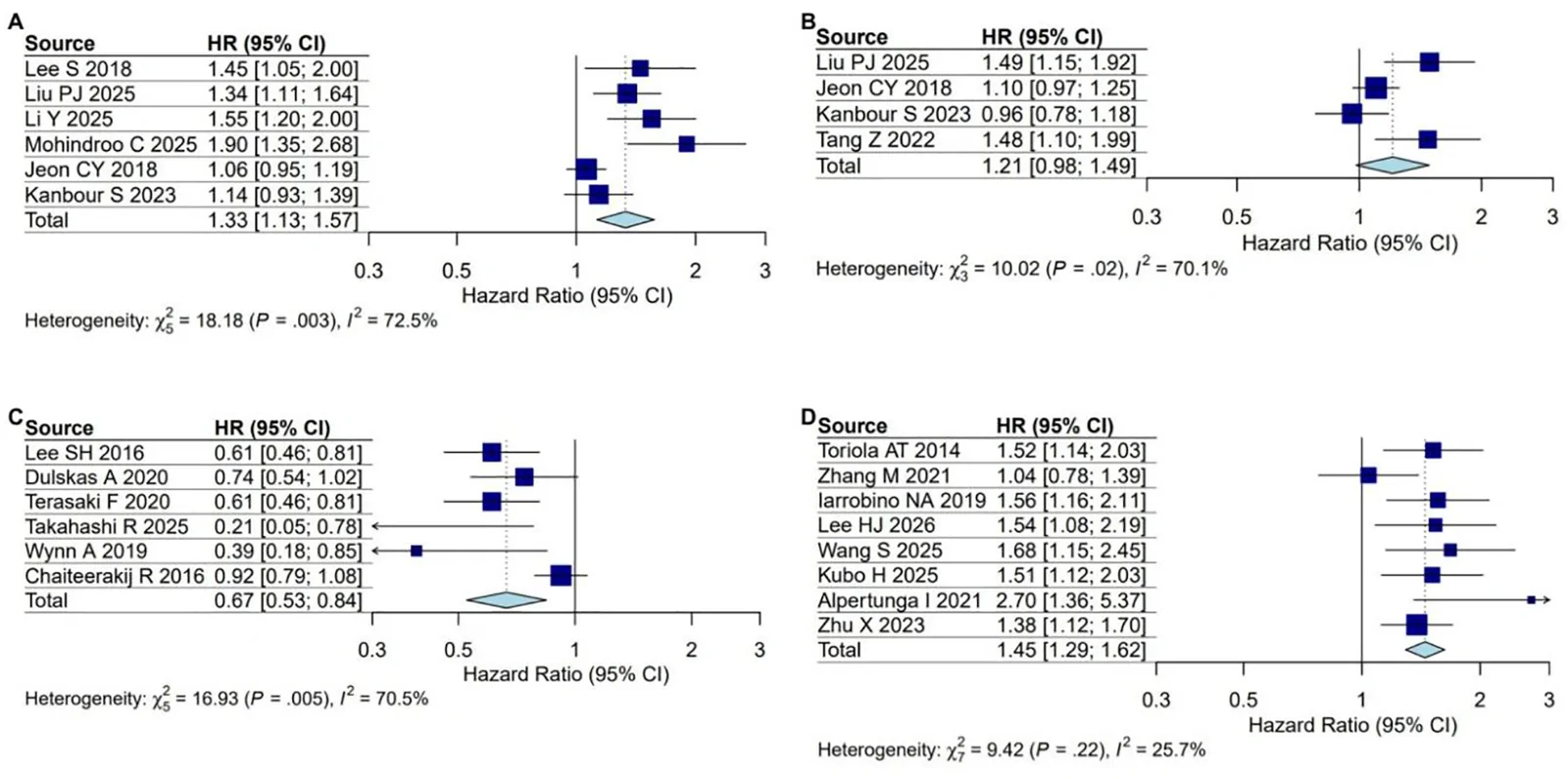
Forest plots of overall survival for the four glycemic exposures. **(A)** New-onset diabetes (NOD). **(B)** Long-standing diabetes (LSD). **(C)** Metformin use. **(D)** Hyperglycemia. The x-axis is shown on a logarithmic scale.

Six studies were pooled (12, 14, 15, 20–22). As shown in **Figure 2A**, NOD was associated with significantly worse OS: pooled HR = 1.33 (95%CI = 1.13–1.57, *p* < 0.001), with high heterogeneity (*I*^2^ = 72.5%, *p* = 0.002). Excluding estimated HRs in a sensitivity analysis did not materially change the estimate (HR = 1.28, 95%CI = 1.10–1.49). Trim-and-fill added zero studies, and the funnel plot showed no obvious asymmetry.

#### Long-standing diabetes *vs*. non-diabetes

Four studies were pooled (12, 13, 20, 21). As shown in **Figure 2B**, LSD was not significantly associated with OS: pooled HR = 1.21 (95%CI = 0.98–1.49, *p* = 0.07), with high heterogeneity (*I*^2^ = 70.1%, *p* = 0.018). No publication bias was detected.

#### Metformin use *vs*. non-use

Six studies were pooled (16, 23–27). As shown in **Figure 2C**, Metformin use was associated with significantly improved OS: pooled HR = 0.67 (95%CI = 0.53–0.84, *p* < 0.001), with high heterogeneity (*I*^2^ = 70.5%, *p* = 0.005). The leave-one-out sensitivity analysis showed that omitting any single study yielded HRs ranging from 0.65 to 0.70, all of which were statistically significant (**Supplementary Table 4a**). To further assess robustness, we performed two additional sensitivity analyses. First, excluding Wynn et al. (16)—the only study with an estimated HR—yielded a pooled HR of 0.68 (95%CI = 0.55–0.85, *I*^2^ = 68.5%), consistent with the primary estimate. Second, using a fixed-effects model produced a similar result (HR = 0.65, 95%CI = 0.55–0.77), confirming that the choice of statistical model did not materially affect the conclusions (**Supplementary Table 4b**). Trim-and-fill added zero studies.

#### Hyperglycemia/poor glycemic control (**Figure 2D**)

Eight studies were pooled (17, 28–34). Hyperglycemia was associated with a 45% increase in mortality: pooled HR = 1.45 (95%CI = 1.29–1.62, *p* < 0.001), with low heterogeneity (*I*^2^ = 25.7%, *p* = 0.22). Trim-and-fill added zero studies, and the funnel plot showed no asymmetry.

#### Indirect comparison between NOD and LSD

To further explore whether diabetes duration modifies prognosis, we performed an indirect comparison using the three studies that reported both NOD and LSD contrasts against the same non-diabetic reference group (Liu et al., Jeon et al., and Kanbour et al.) (12, 20, 21). The pooled HR for NOD *versus* LSD was 1.00 (95%CI = 0.87–1.14, *p* = 0.97), with no evidence of heterogeneity (*I*^2^ = 0%, *p* = 0.38) (**Supplementary Figure 1**). This indirect analysis suggests that, after accounting for the common reference, the survival outcomes of patients with NOD and LSD are not significantly different.

### Publication bias and sensitivity analyses

**Figure 3:**
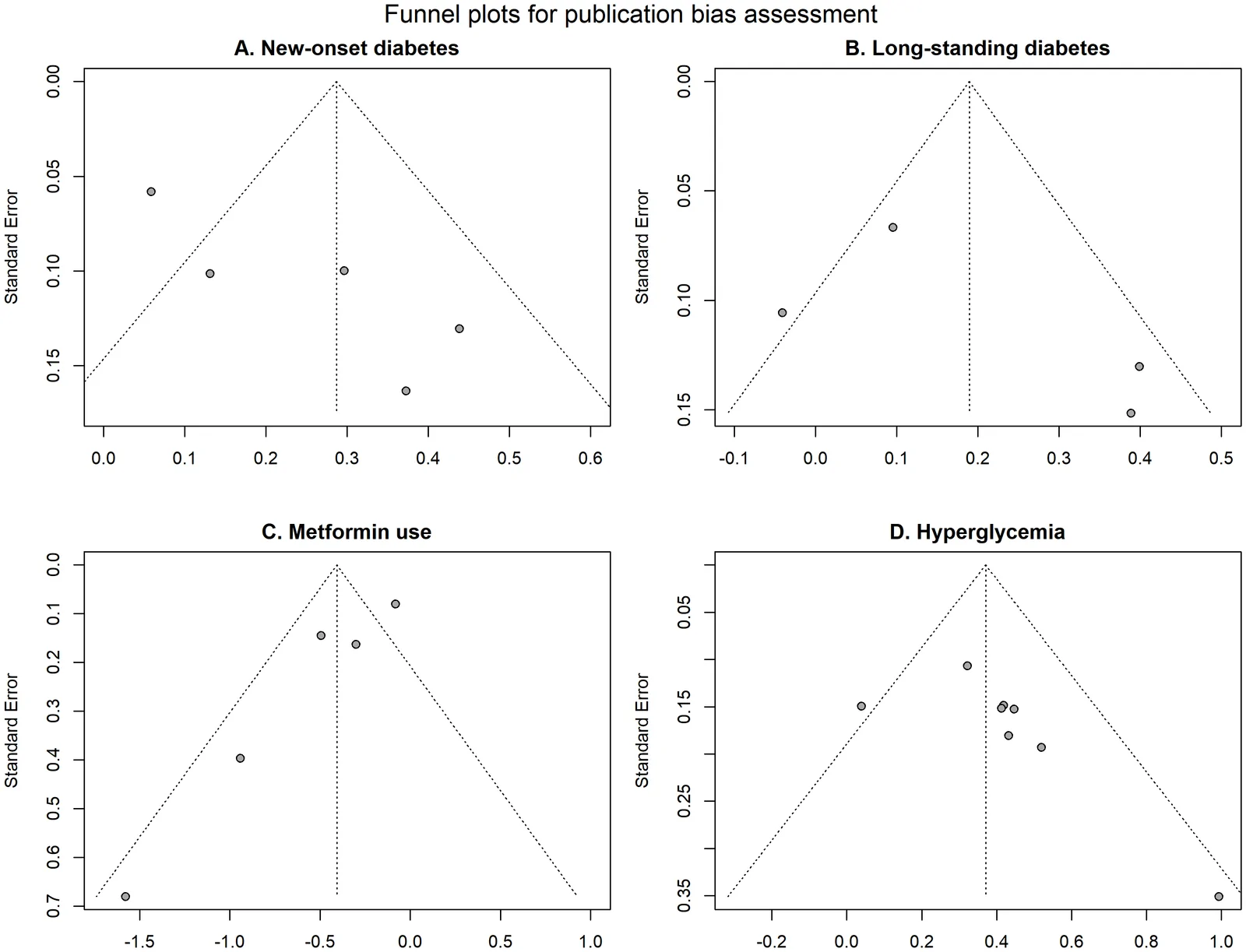
Funnel plots for publication bias assessment: **(A)** NOD, **(B)** LSD, **(C)** metformin use, **(D)** hyperglycemia.

Publication bias was primarily assessed by visual inspection of the funnel plots, which exhibited approximate symmetry for all subgroups (**Figure 3**). Trim-and-fill analysis indicated no missing studies (added studies = 0) for any subgroup. Although Egger’s test was performed for exploratory purposes (see **Table 2**), its results (NOD: *p* = 0.009; metformin: *p* = 0.019) should be interpreted with extreme caution as the test is underpowered and unreliable with fewer than 10 studies per subgroup, according to the recommendations in the Cochrane Handbook. Accordingly, we do not consider these exploratory *p*-values as evidence of substantial publication bias. The metformin subgroup leave-one-out analysis confirmed the robustness of the results (**Supplementary Table 4a**). The leave-one-out analyses for the NOD and hyperglycemia subgroups also showed consistent findings (**Supplementary Tables 5**, **6**). Detailed HR extraction methods for the estimated studies are provided in **Supplementary Table 3**.

**Table 2:** Summary of the subgroup meta-analysis results.

Subgroup	No. of studies	Pooled HR (95%CI)	*p*-value	*I*^2^ (%)	*p* for heterogeneity	Egger’s test *p*	Model
New-onset diabetes *vs*. non-diabetes	6	1.33 (1.13–1.57)	<0.001	72.5	0.003	0.0093	Random
Long-standing diabetes *vs*. non-diabetes	4	1.21 (0.98–1.49)	0.07	70.1	0.02	0.3701	Random
Metformin use *vs*. non-use	6	0.67 (0.53–0.84)	<0.001	70.5	0.005	0.0190	Random
Hyperglycemia *vs*. normoglycemia	8	1.45 (1.29–1.62)	<0.001	25.7	0.22	0.0983	Random

A random-effects model (DerSimonian–Laird) was used for all analyses. Egger’s test was performed for exploratory purposes only. The results are unreliable with <10 studies per subgroup and should not be overinterpreted.

### Certainty of evidence (GRADE assessment)

We assessed the certainty of evidence for each of the four pooled estimates using the GRADE (Grading of Recommendations Assessment, Development and Evaluation) framework (**Supplementary Table 7**). All included studies were observational cohorts, which inherently start at “low” certainty. The evidence was further downgraded for the following reasons:

1. For the NOD *vs*. non-diabetes comparison: high heterogeneity (*I*^2^ = 72.5%) and potential publication bias (exploratory Egger’s test *p* = 0.009, albeit unreliable) led to downgrading for inconsistency and publication bias. The certainty was rated as very low.
2. For the LSD *vs*. non-diabetes comparison: high heterogeneity (*I*^2^ = 70.1%) and imprecision (95%CI crossed the null) led to downgrading for inconsistency and imprecision. The certainty was rated as very low.
3. For metformin use *vs*. non-use: high heterogeneity (*I*^2^ = 70.5%) and potential publication bias (exploratory Egger’s test *p* = 0.019) led to downgrading for inconsistency and publication bias. The certainty was rated as very low.
4. For hyperglycemia *vs*. normoglycemia: low heterogeneity (*I*^2^ = 25.7%) and consistent results across studies led to no downgrading for inconsistency. However, the observational design and the residual confounding risk mean that the evidence starts at “low” certainty and was not upgraded, resulting in a final rating of low.

These GRADE ratings underscore that our findings should be considered hypothesis-generating rather than practice-changing and that prospective studies are urgently needed to confirm these associations.

## Discussion

This meta-analysis, which distinguishes four glycemic exposures, provides several novel insights.

### New-onset diabetes as a negative prognostic marker

NOD was consistently associated with a 33% increase in mortality (HR = 1.33). This finding confirms that NOD is not merely an early diagnostic clue, but is also a marker of aggressive tumor biology (35). The high heterogeneity (*I*^2^ = 72.5%) likely reflects differences in the NOD definitions (e.g., ≤24 months *vs*. ≤12 months), the tumor stage distribution, and the population characteristics. Nevertheless, the direction of effect was uniform across studies, supporting a genuine adverse prognostic impact.

### Long-standing diabetes does not independently affect survival

Pooling four studies, LSD showed no significant association with OS (HR = 1.21, 95%CI = 0.98–1.49), challenging previous beliefs that any diabetes worsens prognosis (36). The high heterogeneity (*I*^2^ = 70.1%) suggests variability in the study populations and adjustment factors. We acknowledge that Tang et al (13). did not specify the diabetes duration, which deviates from our strict LSD definition (>24 months). However, we retained this study for the following reasons: 1) a *post-hoc* sensitivity analysis excluding Tang et al. (13) yielded a nearly identical pooled estimate (HR = 1.15, 95%CI = 0.91–1.45 *vs*. HR = 1.21, 95%CI = 0.98–1.49), indicating that its inclusion does not materially affect the result; 2) the LSD subgroup would otherwise contain only three studies, which would substantially limit the precision and reliability of the pooled estimate; and 3) this limitation is explicitly flagged in **Table 1** (footnote f) and acknowledged in “*Inclusion criteria*.” We therefore considered that retaining this study with full transparency is a reasonable balance between methodological consistency and statistical interpretability.

Our indirect comparison between NOD and LSD (HR = 1.00, 95%CI = 0.87–1.14) suggests that, in this analysis, diabetes duration per se did not further differentiate survival outcomes once diabetes is established. This finding highlights the need for future studies with individual-level data to formally test whether glycemic control—rather than the timing of diabetes onset—mediates the observed association between NOD and worse prognosis. While the similar point estimates for NOD and LSD raise the hypothesis that hyperglycemia may be a key driver, such a mediation effect cannot be confirmed without direct comparison of the glycemic indices between groups.

### Metformin confers survival benefit

Metformin use was associated with a 33% reduction in mortality (HR = 0.67), consistent with preclinical evidence of its anti-proliferative, anti-inflammatory, and immunomodulatory effects (21, 22, 37, 38). The benefit was robust across six studies despite the high heterogeneity (*I*^2^ = 70.5%), which likely reflects differences in metformin exposure timing, dose, and patient selection.

However, this finding should be interpreted with caution. The high heterogeneity (*I*^2^ = 70.5%) and the exploratory Egger’s test *p*-value (0.019, albeit unreliable with *k* < 10) raise concern for potential bias. In addition, some meta-analyses using time-varying Cox models—which better account for immortal time bias—have reported neutral results (39), suggesting that the observed benefit may be partly attributable to methodological artifacts rather than a genuine protective effect. Therefore, we frame this association as hypothesis-generating rather than practice-changing. Well-designed prospective studies or randomized controlled trials are urgently needed to determine whether metformin confers a genuine survival advantage in diabetic PDAC patients. In addition, the definition of metformin exposure varied across studies (e.g., ≥1 month, ≥6 months, preoperative use, or time-varying use), which may have contributed to the observed heterogeneity. Future studies should adopt standardized exposure definitions to facilitate comparability.

### Hyperglycemia is a strong negative prognostic factor

Preoperative hyperglycemia or poor glycemic control was associated with a 45% increase in mortality (HR = 1.45). Notably, the heterogeneity was low (*I*^2^ = 25.7%), indicating consistent results across studies measuring different glycemic indices (fasting glucose, random glucose, or HbA1c). This is clinically actionable: aggressive perioperative glucose management might improve outcomes. Although reverse causation (advanced cancer causing hyperglycemia) cannot be excluded, the consistency across diverse populations and glycemic measures supports a direct detrimental role of hyperglycemia. Mechanistically, hyperglycemia promotes tumor progression through metabolic reprogramming and activation of the insulin/IGF-1 axis (40), and elevated fasting glucose is an independent predictor of cancer-specific mortality in the general population (41). One study in the hyperglycemia subgroup [Alpertunga et al. (17)] defined exposure as RBG-3 >120 mg/dL and reported the HR for good control (HR = 0.37). We inverted this estimate to align with our exposure definition (HR = 2.70). While this inversion is mathematically valid, the glycemic index (random glucose) differs from other studies using fasting blood glucose (FBG) or HbA1c, which may contribute to residual heterogeneity. However, the sensitivity analysis excluding this study did not materially change the pooled estimate (**Supplementary Table 6**).

### Comparison across the four glycemic exposures

Our results highlight that hyperglycemia (HR = 1.45) and NOD (HR = 1.33) are both associated with significantly worse OS, whereas LSD shows a non-significant trend (HR = 1.21). The low heterogeneity in the hyperglycemia subgroup (*I*^2^ = 25.7%) suggests that elevated glucose itself, regardless of the diabetes duration or treatment, is a robust prognostic marker. Importantly, an indirect comparison between NOD and LSD yielded a null result (HR = 1.00), suggesting that, in the pooled analysis, diabetes duration did not further modify survival. This finding should be interpreted as hypothesis-generating. It raises the possibility that glycemic control—rather than the timing of diabetes onset—may be the primary driver of the adverse prognosis associated with NOD. However, formal mediation analysis using patient-level data would be required to confirm this hypothesis. In contrast, the high heterogeneity in the NOD and LSD subgroups underscores the need for standardized definitions and careful adjustment for confounders. The protective effect of metformin (HR = 0.67) is clinically actionable but should be interpreted in light of potential publication bias and residual confounding.

### Comparison with previous meta-analyses

Our findings extend and refine the evidence from earlier meta-analyses that treated diabetes as a single exposure. For instance, a previous meta-analysis (8) reported that diabetes was associated with worse OS in resectable PDAC (HR = 1.44), but did not distinguish NOD from LSD. In contrast, we found that the adverse effect is primarily driven by NOD (HR = 1.33) rather than LSD (HR = 1.21, non-significant). Regarding metformin, another meta-analysis (11) reported a pooled HR of 0.88 (0.80–0.97) for OS, whereas our estimate was 0.67 (0.53–0.84). This discrepancy likely reflects differences in study inclusion: we excluded studies with potential immortal time bias (e.g., a study highlighted in our meta-analysis) (26) and incorporated more recent Asian cohorts where metformin appeared more beneficial. An additional meta-analysis (39) showed that the metformin benefit disappeared when using time-varying Cox models, underscoring the importance of analytic choices. For hyperglycemia, previous meta-analyses have reported a pooled HR of approximately 1.5, which is consistent with our estimate (HR = 1.45). Importantly, no prior meta-analysis has simultaneously quantified the prognostic impact of NOD, LSD, metformin use, and hyperglycemia within a single framework. Thus, our study provides a more nuanced understanding of how distinct glycemic abnormalities influence the prognosis of PDAC and highlights the need for separate clinical consideration of each exposure.

### Clinical implications

Our findings identify several hypotheses that warrant prospective testing. First, patients with newly diagnosed diabetes within 2 years of PDAC diagnosis may represent a subgroup requiring closer surveillance, although the optimal approach remains undefined. Second, while metformin use was associated with survival benefits in the meta-analysis, this finding was based on observational studies with high heterogeneity and potential publication bias; notably, a number of studies using time-varying models have reported neutral results. Therefore, metformin should not be preferentially recommended over other antidiabetic agents based on the current evidence. Third, the consistent association between hyperglycemia and worse survival (*I*^2^ = 25.7%) suggests that rigorous glycemic control may be beneficial, but this also requires confirmation in prospective interventional studies. Until such evidence becomes available, clinical decisions should follow current diabetes management guidelines and consider individual patient characteristics.

### Limitations

All included studies were observational, with an inherent risk of confounding. The small number of studies per subgroup (NOD = 6, LSD = 4, metformin = 6, and hyperglycemia = 8) limited the power of meta-regression and Egger’s test. With fewer than 10 studies per subgroup, formal meta-regression would be statistically underpowered and potentially misleading, as recommended against by the Cochrane Handbook. We therefore did not perform meta-regression. Instead, we addressed heterogeneity through leave-one-out sensitivity analyses, the 24-month definition sensitivity analysis, and GRADE assessment, which explicitly downgrades for inconsistency due to high *I*^2^. The very limited number of studies in the majority of subgroups (*k* ≤ 6) also limits the precision of our random-effects estimates. Alternative estimators such as Hartung–Knapp could not be reliably applied due to the small sample sizes, and our findings should therefore be confirmed in future larger meta-analyses. Some HRs were estimated from survival curves or *p*-values, introducing potential imprecision. Data on diabetes treatment (e.g., insulin or sulfonylureas) were not uniformly available. Fourth, our search was restricted to three databases (PubMed, EMBASE, and Web of Science) and English-language publications. We acknowledge that the English-language restriction may have introduced language bias, as relevant non-English studies—particularly those published in Chinese or Japanese journals, given that 12 of the 21 included studies originated from East Asia—may have been missed. Fifth, we did not search gray literature sources such as conference abstracts, preprints, or clinical trial registries. This may have introduced publication bias by excluding unpublished or non-peer-reviewed data. Although we believe that these limitations are unlikely to have materially altered our main conclusions given the consistency of the findings across studies, we acknowledge their potential impact and have noted this as a limitation. Sixth, despite our efforts to retrieve all potentially eligible full-text articles, 18 reports could not be accessed through institutional subscriptions, open-access searches, interlibrary loan requests, or database access verification. Although these 18 articles represent less than 1% of the initial records identified in the search, their inaccessibility may have introduced a selection bias, and we acknowledge that some relevant data may have been missed. Seventh, although the 2010 start date was chosen to align with the standardized adoption of HbA1c ≥6.5% as a diagnostic criterion, we acknowledge that some relevant studies published between 2000 and 2009 may have been excluded. However, this temporal restriction was deemed necessary to ensure consistent exposure definitions across the included studies. In addition, the majority of studies are from East Asian or North American populations, limiting generalizability to other ethnic groups.

## Conclusions

This meta-analysis demonstrates that NOD and preoperative hyperglycemia are independent negative prognostic factors in pancreatic cancer, whereas LSD is not. However, an indirect comparison showed no significant survival difference between NOD and LSD, raising the hypothesis that glycemic control—rather than diabetes duration per se—may play a more critical role in the prognostic impact of diabetes in PDAC. This hypothesis warrants formal testing in future studies with individual patient data. Metformin use is associated with a significant survival benefit in meta-analyses; however, the strength of this evidence is limited by heterogeneity and publication bias. These findings underscore the importance of distinguishing diabetes subtypes and optimizing glycemic control in patients with PDAC. The potential survival benefit associated with metformin use warrants further investigation in prospective studies, but should not influence current clinical practice based on the available evidence.

## Data Availability

The original contributions presented in the study are included in the article/**Supplementary Material**. Further inquiries can be directed to the corresponding author.
