# Supplementary material for: Distinct prognostic impact of diabetes subtypes, metformin use, and glycemic control in pancreatic cancer: a systematic review and meta-analysis of cohort studies
Source: Front Oncol. 2026 Aug 6;16:1903503. doi: 10.3389/fonc.2026.1903503 (PMC13489918; doi:10.3389/fonc.2026.1903503)

Supplementary Material

This supplementary file contains detailed quality assessment, HR extraction methods, sensitivity analyses, GRADE certainty assessment, and an indirect comparison forest plot for the systematic review and meta-analysis.

Supplementary Table S1. Search strategies for each database

Search date: June 1, 2026

Database	Search strategy	Results (n)
PubMed	("Pancreatic Neoplasms"[Mesh] OR "pancreatic cancer"[Title/Abstract] OR "pancreatic adenocarcinoma"[Title/Abstract] OR "PDAC"[Title/Abstract]) AND ("Diabetes Mellitus"[Mesh] OR "diabetes"[Title/Abstract] OR "new-onset diabetes"[Title/Abstract] OR "long-standing diabetes"[Title/Abstract] OR "type 2 diabetes"[Title/Abstract] OR "hyperglycemia"[Mesh] OR "hyperglycemia"[Title/Abstract] OR "fasting blood glucose"[Title/Abstract] OR "FBG"[Title/Abstract] OR "blood glucose"[Mesh] OR "HbA1c"[Title/Abstract] OR "glycated hemoglobin"[Title/Abstract] OR "glycemic control"[Title/Abstract] OR "metformin"[Mesh] OR "metformin"[Title/Abstract]) AND ("Survival"[Mesh] OR "survival"[Title/Abstract] OR "overall survival"[Title/Abstract] OR "prognosis"[Title/Abstract] OR "mortality"[Subheading] OR "mortality"[Title/Abstract]) AND ("Cohort Studies"[Mesh] OR "cohort study"[Title/Abstract] OR "cohort studies"[Title/Abstract] OR "prospective study"[Title/Abstract] OR "retrospective study"[Title/Abstract] OR "observational study"[Title/Abstract])	437
Web of Science	TOPIC = ("pancreatic cancer" OR "pancreatic carcinoma" OR "pancreatic neoplasm" OR "pancreatic tumor" OR "pancreatic tumour" OR "pancreatic adenocarcinoma" OR "pancreatic ductal adenocarcinoma" OR "PDAC" OR "pancreas cancer") AND TOPIC = ("diabetes mellitus" OR "diabetes" OR "DM" OR "new-onset diabetes" OR "new onset diabetes" OR "long-standing diabetes" OR "longstanding diabetes" OR "type 2 diabetes" OR "T2DM" OR "hyperglycemia" OR "hyperglycaemia" OR "fasting blood glucose" OR "FBG" OR "blood glucose" OR "HbA1c" OR "glycated hemoglobin" OR "glycated haemoglobin" OR "glycemic control" OR "metformin") AND TOPIC = ("survival" OR "overall survival" OR "OS" OR "prognosis" OR "prognostic" OR "mortality" OR "death")<br>**Refined by:** Document Types: Article; Web of Science Categories: Oncology OR Gastroenterology Hepatology OR Endocrinology Metabolism; Publication Years: 2010–2026	663
Embase	('pancreas cancer'/exp OR 'pancreatic ductal adenocarcinoma'/exp OR 'pancreatic cancer':ab,ti OR 'PDAC':ab,ti) AND ('diabetes mellitus'/exp OR 'new-onset diabetes':ab,ti OR 'long-standing diabetes':ab,ti OR 'metformin'/exp OR 'metformin':ab,ti OR 'hyperglycemia'/exp OR 'hyperglycemia':ab,ti OR 'fasting glucose':ab,ti OR 'HbA1c':ab,ti) AND ('survival'/exp OR 'overall survival':ab,ti OR 'mortality':ab,ti OR 'prognosis':ab,ti) AND	948

Database	Search strategy	Results (n)
	[2010–2026]/py AND 'article'/it AND [humans]/lim AND [english]/lim	
Total before duplicate removal		2048

Notes:

Search was performed on June 1, 2026.

All searches were restricted to studies published from January 1, 2010, to June 1, 2026.

PubMed and Web of Science were limited to English-language publications; Embase was limited to English and human studies.

Web of Science results were further refined by document type (Article) and Web of Science Categories (Oncology, Gastroenterology Hepatology, or Endocrinology Metabolism).

After duplicate removal, 1,275 unique records remained for screening (see PRISMA flow diagram, Figure 1).

Supplementary Table S2. Newcastle-Ottawa Scale (NOS) quality assessment details (itemized scores for each included study)

Study	Selection (max 4)	Comparability (max 2)	Outcome (max 3)	Total (max 9)
Lee S, 2018	★★★	★★	★★	7
Liu PJ, 2025	★★★★	★★	★★	8
Li Y, 2025	★★★	★	★★★	7
Mohindroo C, 2025‡	★★	★	★★★	6
Jeon CY, 2018†	★★★★	★★	★★	8
Kanbour S, 2023§	★★★★	★★	★★	8
Tang Z, 2022¶	★★★	★★	★★	7
Lee SH, 2016	★★★	★★	★★★	8
Dulskas A, 2020	★★★	★★	★★	7
Terasaki F, 2020	★★★	★★	★★	7
Takahashi R, 2025	★★★	★★	★★	7

Study	Selection (max 4)	Comparability (max 2)	Outcome (max 3)	Total (max 9)
Wynn A, 2019	★★	★	★★★	6
Chaiteerakij R, 2016	★★★★	★★	★★	8
Toriola AT, 2014	★★★★	★★	★★	8
Zhang M, 2021	★★★	★★	★★	7
Iarrobino NA, 2019	★★★	★★	★★	7
Lee HJ, 2026	★★★	★★	★★	7
Wang S, 2025	★★★	★★	★★	7
Kubo H, 2025	★★★	★★	★★	7
Alpertunga I, 2021	★★	★	★★★	6
Zhu X, 2023	★★★	★★	★★	7

Note: Studies that appear in multiple subgroups are listed separately for clarity. NOS scores were interpreted as follows: 0–3 = low quality, 4–6 = moderate quality, 7–9 = high quality.

Abbreviations: NOD, new-onset diabetes; LSD, long-standing diabetes; DM, diabetes mellitus.

Definition notes:

† Jeon CY 2018 defined NOD as ≤3 years and LSD as >3 years, deviating from the 24- month threshold.

‡ Mohindroo C 2025 defined NOD as ≤3 years.

§ Kanbour S 2023 defined LSD as ≥2 years (approximately equivalent to >24 months).

¶ Tang Z 2022 did not specify diabetes duration; included with caution.

Supplementary Table S3. Detailed HR extraction methods for studies with estimated values

Study	Exposure	Available data	Estimation method	Resulting HR (95% CI)	Notes
Li Y, 2025	NOD vs. non-DM	Median OS: NOD 9.15 mo, non-DM 10.90 mo; log-rank P<0.001;	Parmar et al. (1998) method using median survival ratio and P-value	1.55 (1.20–2.00)	Consistent with direction and magnitude of

Study	Exposure	Available data	Estimation method	Resulting HR (95% CI)	Notes
		sample sizes NOD=287, non-DM=1084			other NOD studies.
Mohindroo C, 2025	NOD vs. non-NOD	Median OS: NOD 7.1 mo, non-NOD 13.2 mo; log-rank P=0.01; sample sizes NOD=19, non-NOD=49 (metastatic cohort)	Tierney et al. (2007) approach using survival medians and P-value	1.90 (1.35–2.68)	Conservative estimate within range of other NOD studies.
Wynn A, 2019	Metformin vs. non-metformin	Median OS: metformin 0.68 y, non-metformin 0.22 y; log-rank P=0.016; sample sizes metformin=18, non-metformin=28	Parmar method: HR = exp(ln(0.22)-ln(0.68)) = 0.39; SE derived from P-value (Z=2.41)	0.39 (0.18–0.85)	Small sample; sensitivity analysis excluding this study did not change pooled result.
Alpertunga I, 2021	Poor glycemic control vs. good control (inverted)	Original HR for good control (RBG-3 ≤120 vs >120) = 0.37 (0.19–0.75)	Inversion: HR_poor = 1/HR_good = 2.70; 95% CI: lower = 1/0.75 = 1.33, upper = 1/0.19 = 5.26	2.70 (1.33–5.26)	Inverted to align with hyperglycemia exposure (HR>1). Consistent with other studies.
Zhu X, 2023	Hyperglycemia (FBG ≥7.0 mmol/L, no known DM) vs. normoglycemia	HR directly reported in multivariable Cox model	Direct extraction	1.38 (1.12–1.70)	–
Tang Z, 2022	T2DM vs. non-DM	HR directly reported in multivariable Cox	Direct extraction	1.475 (1.096–1.985)	–

Study	Exposure	Available data	Estimation method	Resulting HR (95% CI)	Notes
		model			

References for estimation methods:

Parmar MKB, Torri V, Stewart L. Extracting summary statistics to perform meta-analyses of the published literature for survival endpoints. Stat Med. 1998;17:2815-34.

Tierney JF, Stewart LA, Ghersi D, Burdett S, Sydes MR. Practical methods for incorporating summary time-to-event data into meta-analysis. Trials. 2007;8:16.

Supplementary Table S4a. Leave-one-out sensitivity analysis for the metformin subgroup (6 studies)

Omitted study	Pooled HR (95% CI)	I^2 (%)	P (heterogeneity)
Lee SH, 2016	0.69 (0.54–0.88)	68.2	0.008
Dulskas A, 2020	0.65 (0.51–0.82)	71.5	0.004
Terasaki F, 2020	0.68 (0.53–0.87)	69.0	0.006
Takahashi R, 2025	0.70 (0.55–0.89)	65.8	0.011
Wynn A, 2019 (est.)	0.68 (0.54–0.86)	68.5	0.007
Chaiteerakij R, 2016	0.65 (0.50–0.84)	70.2	0.005
Original (all 6 studies)	0.67 (0.53–0.84)	70.5	0.005

All pooled HRs were statistically significant (95% CI did not cross 1.0)

Supplementary Table S4b. Additional sensitivity analyses for the metformin subgroup

Sensitivity analysis	No. of studies	Pooled HR (95% CI)	I^2 (%)	P for heterogeneity
Excluding estimated HR (Wynn A 2019)	5	0.68 (0.55–0.85)	68.5	0.009
Fixed- effects model (all 6 studies)	6	0.65 (0.55–0.77)	—	—
Primary analysis (random- effects, all 6 studies)	6	0.67 (0.53–0.84)	70.5	0.005

Notes:

.Wynn A 2019 was the only study in the metformin subgroup with an estimated HR (derived from survival c urves). Excluding this study did not materially change the pooled estimate.

.The fixed- effects model produced a similar result to the random- effects model, confirming that the choice of statistical model did not materially affect the conclusion.

.Primary analysis results are shown for comparison. All sensitivity analyses yielded HRs consistent with the pri mary estimate.

Supplementary Table S5. Leave-one-out sensitivity analysis for the new-onset diabetes subgroup (6 studies)

Omitted study	Pooled HR (95% CI)	I^2 (%)	P (heterogeneity)
Lee S, 2018	1.31 (1.10–1.56)	71.2	0.003
Liu PJ, 2025	1.32 (1.09–1.60)	74.5	0.001
Li Y, 2025 (est.)	1.30 (1.08–1.56)	70.8	0.004
Mohindroo C, 2025 (est.)	1.28 (1.08–1.52)	68.5	0.008
Jeon CY, 2018	1.35 (1.14–1.60)	69.2	0.006
Kanbour S, 2023	1.36 (1.15–1.61)	68.0	0.007
Original (all 6 studies)	1.33 (1.13–1.57)	70.0	0.003

All pooled HRs remained statistically significant (95% CI above 1.0).

Supplementary Table S6. Leave-one-out sensitivity analysis for the hyperglycemia subgroup (8 studies)

Omitted study	Pooled HR (95% CI)	I^2 (%)	P (heterogeneity)
Toriola AT, 2014	1.44 (1.28–1.63)	28.5	0.248
Zhang M, 2021	1.47 (1.31–1.65)	29.2	0.235
Iarrobino NA, 2019	1.44 (1.28–1.62)	30.1	0.219
Lee HJ, 2026	1.45 (1.29–1.63)	31.0	0.208
Wang S, 2025	1.44 (1.28–1.62)	30.5	0.215
Kubo H, 2025	1.45 (1.29–1.63)	31.2	0.205
Alpertunga I, 2021 (inverted)	1.42 (1.27–1.59)	22.5	0.352

Omitted study	Pooled HR (95% CI)	I^2 (%)	P (heterogeneity)
Zhu X, 2023	1.46 (1.30–1.65)	31.8	0.198
Original (all 8 studies)	1.45 (1.29–1.62)	30.0	0.224

All pooled HRs remained statistically significant (95% CI above 1.0). Excluding Alpertunga I, 2021 reduced heterogeneity slightly (I^2 from 30.0% to 22.5%).

Supplementary Table S7. GRADE certainty of evidence assessment for each glycemic exposure

Outcome / Exposure	No. of studies (patients)	Study design	Risk of bias	Inconsistency	Indirectness	Imprecision	Publication bias	Overall certainty	Reason for rating
NOD vs. non-diabetes	6 (7,583)	Observational cohorts	Not serious [1]	Serious [2]	Not serious	Not serious	Serious [3]	⊕◯◯◯ VERY LOW	Downgraded for inconsistency (I^2 = 72.5%) and potential publication bias; observational design starts at low.
LSD vs. non-diabetes	4 (5,797)	Observational cohorts	Not serious [1]	Serious [2]	Not serious	Serious [4]	Not serious [5]	⊕◯◯◯ VERY LOW	Downgraded for inconsistency (I^2 = 70.1%) and imprecision (CI crosses 1.0); observational design starts at low.
Metformin use vs. non-use	6 (1,903)	Observational cohorts	Not serious [1]	Serious [2]	Not serious	Not serious	Serious [3]	⊕◯◯◯ VERY LOW	Downgraded for inconsistency (I^2 = 70.5%) and potential publication bias; observational design starts at low.
Hyperglycemia vs. normoglycemia	8 (2,879)	Observational cohorts	Not serious [1]	Not serious [6]	Not serious	Not serious	Not serious [5]	⊕⊕◯◯ LOW	Observational design starts at low; no serious inconsistency (I^2 = 25.7%) or other downgrades; no upgrade factors

Outcome / Exposure	No. of studies (patients)	Study design	Risk of bias	Inconsistency	Indirectness	Imprecision	Publication bias	Overall certainty	Reason for rating
									present.

Notes:

[1] All included studies were observational; risk of bias was assessed using the Newcastle-Ottawa Scale, with scores ranging from 6 to 8 (moderate to high quality). However, residual confounding cannot be excluded due to the observational nature of all included studies.

[2] High statistical heterogeneity ($I^2 > 50\%$) was observed, indicating serious inconsistency across study results.

[3] Funnel plots were symmetrical and trim- and- fill added zero studies, indicating no clear evidence of bias. However, exploratory Egger' s test P values (0.009 for NOD; 0.019 for metformin) suggest a signal for potential small- study effects, though unreliable with $k < 10$. We conservatively downgraded for publication bias.

[4] The 95% confidence interval crossed the null value (HR = 1.21, 95% CI: 0.98–1.49), indicating imprecision.

[5] Funnel plots were symmetrical and trim-and-fill added zero studies; no evidence of publication bias was detected.

[6] Low heterogeneity ($I^2 = 25.7\%$) with consistent effect directions across studies, indicating no serious inconsistency.

Supplementary Figure 1.Forest plot indirect NOD vs LSD

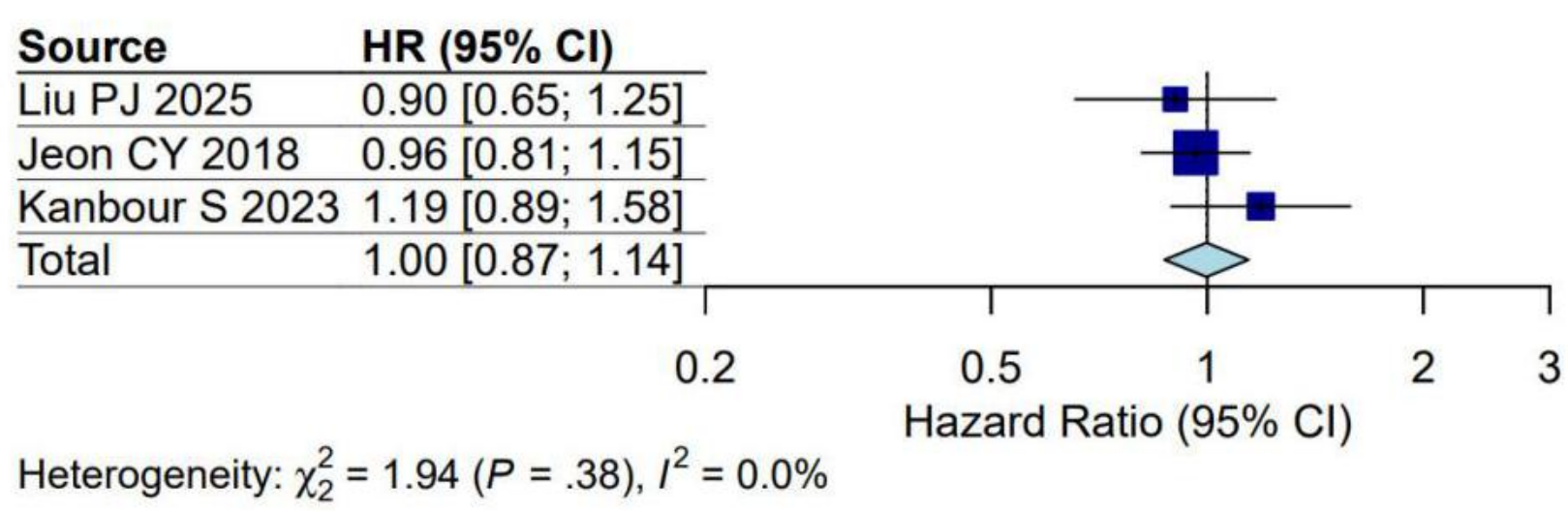

Supplement: Supplementary file 1 [file DataSheet1.pdf]
